# Social and Cognitive Psychology Theories in Understanding COVID-19 as the Pandemic of Blame

**DOI:** 10.3389/fpsyg.2021.672395

**Published:** 2022-01-13

**Authors:** Ayoub Bouguettaya, Clare E. C. Walsh, Victoria Team

**Affiliations:** ^1^School of Psychology, University of Birmingham, Birmingham, United Kingdom; ^2^Independent Researcher, Birmingham, United Kingdom; ^3^Monash Nursing and Midwifery, Level 5 Alfred Centre, Melbourne, VIC, Australia

**Keywords:** COVID-19, blame, social identity, social cognition, pandemics and epidemics, social psychology, Path Model of Blame

## Abstract

When faced with adverse circumstances, there may be a tendency for individuals, agencies, and governments to search for a target to assign blame. Our focus will be on the novel coronavirus (COVID-19) outbreak, where racial groups, political parties, countries, and minorities have been blamed for spreading, producing or creating the virus. Blame—here defined as attributing causality, responsibility, intent, or foresight to someone/something for a fault or wrong—has already begun to damage modern society and medical practice in the context of the COVID-19 outbreak. Evidence from past and current pandemics suggest that this tendency to seek blame affects international relations, promotes unwarranted devaluation of health professionals, and prompts a spike of racism and discrimination. By drawing on social and cognitive psychology theories, we provide a framework that helps to understand (1) the effect of blame in pandemics, (2) when people blame, whom they blame, and (3) how blame detrimentally affects the COVID-19 response. Ultimately, we provide a path to inform health messaging to reduce blaming tendencies, based on social psychological principles for health communication.

## Introduction

Blame is a feature of individual, organizational, system and government responses to COVID-19 pandemic worldwide. Struggling to deal with an invisible, organic threat, many governments, agencies, and individuals have sought instead to assign undue responsibility of the spread of COVID-19 to groups and entire countries (e.g., China; Al-Jazeera, 2020), minority groups (Sarkar, 2020; Markowitz et al., 2021). The World Health Organization has suggested that the language used around pandemics is critical to limiting blame and stigma, but many world leaders have paid no heed to this advice, calling COVID-19 by regional language or variants by their location of origin (World Health Organisation [WHO], 2020). For example, former US president Donald Trump repeatedly called the virus the “China Virus” to blame China for the spread of COVID-19, even crossing out “COVID-19” on his script (Smith, 2020). Blame was also directed by agencies against governments; for example, the Brazilian Education minister blamed China for COVID-19 as a plan for “world domination” (Al-Jazeera, 2020). Assigning blame to specific groups and agencies (sometimes unduly) during COVID-19 appears to be common in many countries (Montiel et al., 2021), at multiple levels (e.g., Australia’s blame game between media, state, federal, and local governments; see Hoffman et al., 2020). Recent research also suggests that the blame-game behavior may negatively affect compliance with public health directives (Stadler, 2003; Mahajan et al., 2008).

Blame can have wide ranging consequences, both directly on pandemic response and indirectly through influencing undesirable and unacceptable general social issues. Blame is, simply put, when one individual/group attributes responsibility, intent, foresight, or causality to another individual/group for an event (Malle et al., 2014). Historically, minority groups were blamed for pandemics with deadly consequences. Jewish communities were blamed for the Black Death pandemic in the 1300’s. Consequently, thousands of Jewish people were killed (Zahler, 2009). Sexual health epidemics were blamed on other countries. Syphilis was called “the French disease” in Italy, and “the Italian disease” in France (Cassar, 2002). There exists a long history of blaming “others” for diseases in more recent times, such as the “Mexican Swine Flu” in 2009 (Cohn, 2012; Habicht et al., 2020).

This effect of blame has direct negative consequences on managing pandemics. Because blame can cause stigma, individuals from blamed groups may conceal or hide their illness (Dar et al., 2020; Singh and Subedi, 2020). Multiple studies have found blaming individuals or groups for HIV/AIDS leads to stigma, which led to weaker intentions to seek treatment, or conceal their illness (Stadler, 2003; Mahajan et al., 2008). In more recent news, in Iran, the stigma of having COVID-19 in the house was so great that if a case showed up at home, the head of the household could be blamed for failing to protect their family. As a result of this, patients hid their illness, and COVID-19 spread extremely quickly in Iran compared to its neighbors. This likely led to a severe undercounting of deaths (Asadi-Aliabadi et al., 2020; Rubin, 2020).

Research on COVID-19 and blame has highlighted some trends in how blame operates and spreads. An analysis of 1 million instances of Chinese online material (including Facebook texts and news), found information on these pages frequently spread information that blamed China or Wuhan residents for the spread of COVID-19 (Chang et al., 2020). Another multilingual text analysis of online platforms found that blaming specific agents for COVID-19 comprised of 15% of online texts, with most being false (Islam et al., 2020). Further experimental research on Americans found that conservatives were more likely than liberals to blame Democrats, Republicans, Chinese people, and the Chinese government for COVID-19’s impact in the United States (Porumbescu et al., 2020). When exposed to the term “China-virus,” all participants of the study became more likely to blame Chinese residents (Porumbescu et al., 2020). While these studies have shown that certain people are more likely to blame certain targets, and that blame can spread quickly, these studies say little on the mechanics of blame in COVID-19.

Surprisingly, at time of writing, little direct psychological research has been done on blame and COVID-19 beyond commentary; a cursory search on this topic revealed only four papers that sought to understand blame in COVID-19. This may be because psychological research on the factors of blame (both theoretical and empirical) has yet to be adopted by the wider medical and health community. Understanding the psychology of blame may help inform an agenda on reducing blame in COVID-19 and improve COVID-19 risk communication and the outbreak management response in present and future contexts. Here, we describe how blame has affected the pandemic response, leading to a discussion on the psychology of blame (when and who people blame), how blame works to weaken the response, and how we can reduce blame in COVID-19. We consolidate a socio-cognitive model of blame (Malle et al., 2014) and social identity research (Jetten et al., 2020) to inform a model of blame in COVID-19.

## When Do People Blame?

Attribution models (including blame) have been a mainstay of psychology and anthropology for decades (Heider, 1958; Kelley, 1973). Older Freudian perspectives on blame famously suggested it was a defense mechanism to shift responsibility onto others to protect one’s ego (Freud, 1946). As such, Freud suggested that “blame projection” was an immature defense mechanism; later psychodynamic research suggested that certain people were more likely to employ blame projection when they were less trusting than others (Hochreich, 1975).

A more recent theory of blame (the Path Model of blame) suggests that blame is cognitive, social, and requires warrant (Malle et al., 2014). The model posits that blame comes in a private, cognitive form based on one’s characteristics and social cognition, and a public social form where the blame is guided by a set of norms, and roles designed to regulate community and social relationships. This model suggests a first step, where the perceiver considers whether a particular agent or target caused an event or outcome that violated a social norm. Then, if there was clear intent, blame is allocated, but if there was no intent, responsibility and capacity to prevent the issue are considered (Malle et al., 2014). This model suggests blame is most likely to occur under these circumstances, but also elaborates on *what* events cause blame to extrapolate “when.” The events have to be detectable as a norm violation. For example, aged care workers in Australia were blamed for spreading COVID-19 in aged care homes; this can easily be considered to be a norm violation (Team and Manderson, 2020). However, people may differ in terms of the intent attribution as a function of *which* norm is subjectively being violated, and differ in their views of responsibility and preventative capacity. Specifically, one person may blame just the infected person as they would believe the norm of individual responsibility. Another person may instead consider responsibility and capacity of others (e.g., aged care homes and the government) to prevent these things from occurring through providing proper training and protective materials in aged care homes. The social component in this model is particularly pertinent, therefore, in understanding blame in a pandemic (Malle et al., 2014).

## Blame as a Social Phenomenon: Who Is Blamed, and What Factors Affect Blame?

Because blame targeting is largely a social phenomenon, understanding the social goals and norms that guide this behavior and cognition from an established framework will be requisite. The social identity approach is one such approach that has detailed how norms are a function of intergroup dynamics (Abrams and Hogg, 1990). This approach posits that people join groups to feel good and special (i.e., as part of a drive for positive distinctiveness), and have a drive to maintain the positive distinctiveness of their group by ensuring their group is *better* than other groups (Tajfel, 1974a; Tajfel et al., 1979). This drive leads intergroup relations, and affects how groups interact with one another (Tajfel, 1974b). One particular intergroup context could potentially lead to greater blame: when one group, of a higher status than another, maintains higher status by blaming a lower status group for a given problem. Admission of failure may reduce the positive distinctiveness individuals normally would get from their group membership, and blame reduces this sensation as it redirects the responsibility away from their group to another. For example, if a person is a strong identifier with the Conservative party in the United Kingdom (i.e., a member of the party), and the Conservative leadership fails to secure enough protective equipment for healthcare workers, that violates the embedded norm of “Britain first” that the party espouses (see Reicher et al., 2005; Hansson, 2019 for a discussion on social identity and leadership). In this context, individuals can either acknowledge the failures of their party (weakening the positivity of their group), or allocate blame to another group, like the EU or China.

Overall, a novel socio-cognitive integrative framework of blame for when blame occurs and who is blamed can be created from this social and cognitive evidence. First, from a social perspective, the purpose of blame is a form of diffusion of responsibility in order to maintain one group’s status relative to others and regulate the behavior of ingroup and outgroup members. This affects *who* is blamed. The cognitive component affects *when* blame is used as a regulation strategy; this is where individuals must consider warrant and the actual information used to make this assessment. The blamer must have information on the intent, causality, and preventability of the event that clearly can be used to justify the blame. In this integrative framework, social groups provide direction and drive, while cognition gives rationality behind blame (allowing for justification). In the previous example, because the Conservative party member is driven toward positive distinctiveness, they are driven to choose to blame another group, and the group they select must make their group look good by comparison (Krylova et al., 2017). This means that they may choose the EU, a group that has a strained relationship with the United Kingdom since Brexit. Their ability to rationalize blame would be dependent on their cognition; here, the conservative member might ascribe intent (e.g., “they chose to withhold supplies”) or responsibility and capacity (e.g., “they knew this would happen, and they could have helped but didn’t”).

There are some situations where blame can be helpful, such as a retrospective tribunal examining where fault lies in order to improve systems or existing responses to emergent issues, such as COVID-19. For example, the EU has a commission that seeks to examine where the failures are COVID-19 containment and rectify them (EU Directorate-General for Communication, 2021). This is to say that blame is sometimes warranted; there is a strong case to be made for some leaders, political parties, groups, and individuals failing to protect the public from COVID related consequences. For example, there is evidence that the UK government’s “Eat out to help out scheme,” which gave cash for people to eat inside restaurants likely accelerated the second wave of COVID-19 cases (Fetzer, 2020) and blaming the government for this failure would likely be warranted. However, often blame takes the form of assigning responsibility or intent to individuals or groups that have no role in the problem, or assign blame too early for it to be of use. In some cases, governments can be blamed no matter what they do; for example, the Australian government was blamed for failures for repatriating flights from India when Delta arose in the country, but was also blamed for Delta coming to Australia after the ban was lifted (Gunia, 2021). As we are writing this in the middle of the pandemic and data on the key elements of blame (responsibility, evidence, foresight) is scant, we will not distinguish between due and undue blame here.

Because blame requires warrant (being able to provide evidence in the form of causality, intent, and preventability), and the social drive to maintain positive distinctiveness is so strong, creative solutions to creating warrant may be used instead (Greene et al., 2020). This means using moral grounds to establish blame, which can result in undue blame targeted against a group or individuals that have little to do with the issue or problem. Because morality is a function of one’s social group (Ellemers et al., 2013; Parker and Janoff-Bulman, 2013), this means that the evidence used may not actually make sense to an outgroup member, which in turn may increase animosity. When the response to a crisis requires a co-ordinated response, blame can be toxic. For example, the former president Donald Trump’s tendency to blame China for COVID-19 (which also occurred in the middle of a trade war) resulted in worse relations when Chinese manufacturing was essential to deliver medical equipment (Tan, 2020). Blame games within the United States on COVID-19 supplies also did not help with the response between federal and state agencies (Forester and McKibbon, 2020). The virtual G7 and G20 summits were an exercise in blame shifting as well; instead of a collaborative response, it devolved into an argument on who to blame, and the United States even blocked a statement on the leadership role of the World Health Organization as a result of this disagreement (Forman et al., 2020).

## How Blame Reduces Effective Responses to a Pandemic in the Community

Blame can lead to divisions in the international community in a pandemic (e.g., the United States and China), but blame can lead to divisions within the community when a full community response is required (Jetten et al., 2020). When a minority group is blamed for a pandemic, the social identity approach would argue that this means the pandemic is no longer a problem of *we* but rather *them* (Tajfel, 1974b; Tajfel et al., 1979). When a pandemic, such as COVID-19, requires voluntary responses for the collective, this blame can be damaging for the willingness of the subgroups to engage with government services and directives. In India, for example, the population was directed by the government to quarantine in response to COVID-19. However, in response to an outbreak of COVID-19 that occurred due to Islamic gatherings, MPs from the nationalist Hindu ruling party (the BJP), used this to blame Muslims as super-spreaders, calling it “corona terrorism” (Ellis-Petersen and Rhaman, 2020). Muslims in India were then ostracized, and ostensibly, may have concealed their symptoms rather than get help. Similarly, ethnoreligious minorities in the United Kingdom were blamed for the spread of COVID-19 by a member of the ruling party—and the prime minster, Boris Johnson, did not condemn these comments (Ellis-Petersen and Rhaman, 2020).

Post-COVID-19, blame can also lead to significant fractures in intergroup cooperation for future threats. It is evident that blaming China for the COVID-19 pandemic will have long standing consequences. China has already retaliated against Australia for blaming China for COVID-19’s origins, by placing tariffs on Australian goods (British Broadcasting Corporation [BBC], 2020a). Tensions in India between Hindus and Muslims are already strained, and COVID-19 fractures may worsen these relations (Sarkar, 2020). Historical evidence suggests that pandemic blaming can even cause long lasting effects; for example, the cholera epidemics in early nineteenth century America and Europe led to several riots against doctors, hospital workers, and government workers, which contributed to distrust of the government for decades (Rosenberg, 2009). Blaming the Church for the Black Death in Europe may have helped hasten its downfall as well, as it has been argued the failure of the church to deal with the pandemic shook people’s confidence in the clergy and the power of the church (Zentner, 2015). It is likely that blame in pandemics, such as in COVID-19, will have similarly severe consequences on intragroup and intergroup functioning.

## How to Reduce Blame in a Pandemic

Blame in a pandemic is not necessarily an instinctive response, but rather a manufactured one that relies on social norms above all else; the level of blame appears to be dependent on the greater context in which pandemics occur (Cohn, 2012). In fact, in antiquity, many pandemics resulted in communities working together, rather than blame (Cohn, 2012)—but this largely only happened if the community had an effective response that maintained social structures (Habicht et al., 2020). As stated earlier, blame can help to reduce responsibility from one’s own group to another, so combating blame while maintaining a positive social standing can be difficult.

There are two main ways that have been proposed to reduce blame. The first method comes from political science and law. A recent paper with seven studies and agent-based modeling suggested that the best thing to do to reduce blame is to focus on praising as many people as possible on success, and blame as narrowly as possible after failure (Schein et al., 2020). In the context of COVID-19, this would mean focusing as much as possible on the people who have done the *right* thing and the successes along the way, and blame should be used extremely rarely to met out judgment on very narrow targets (e.g., a failed health minister who violated social norms for their own gain). This method, in Path Based Model of blame, would work by changing *when* blame is used.

The other way to reduce blame in COVID-19 has been discussed (albeit indirectly) in a recent social identity analysis of COVID-19 (Jetten et al., 2020). Leaders and health professionals must ensure their messages on COVID-19 unite, rather than to divide by fostering a sense of “us” above all else. It may be politically expedient to blame particular groups, but ultimately it not only damages the response by causing those groups not to comply, but also potentially may lead to future problems in intragroup relations (Jetten et al., 2020). Theoretically, this will instill social norms that focus less on individual responsibility (i.e., “those bad rule breakers”) and rather ones of shared, collective responsibility (i.e., “we’re in this together”), changing *who* is blamed.

Overall, these streams of research suggest that the best way policy makers can act to reduce blame is through harnessing social identification for good: *protect us, because that’s what we do*. Focusing on others may not be helpful. Instead, messaging about *us* doing the *right* thing is key. Recent evidence showed that social identification is a consistent predictor of health behaviors several months later, meaning harnessing this power of us is useful (Cárdenas et al., 2021). Another paper provides evidence that that family, community, and national identification has significant links with self-reported helping and physical distancing, and provides an example of good health messaging around these topics (Vignoles et al., 2021). Similarly, public health messages around protecting us are more powerful than protecting oneself (Wang and Lee, 2020; Gerber et al., 2021), as these messages can build trust between ethnic groups and governments (Razai et al., 2021).

Despite this evidence, there is little experimental research on the effects of blame messages on people’s health behavior in the context of COVID-19. Most research focuses on messages designed to build community solidarity or correlational research on social identity and (Vignoles et al., 2021), but there is no experimental evidence on the effect of blame type messages weakening a response or intentions compared to non-blame messages. It is possible that a fine-tuned collective based blame messages on outgroups (using the Path Model of Blame Malle et al., 2014), coupled *with* messages on us as a contrast, may actually improve adherence to public health directives. One such example would be to say that *we* take care of each other, even though it’s *them* that caused it, *we* can fix it. From a social identity approach, this is theoretically plausible; social identity content (who we are) is partially defined by what we are not, and harnessing this may be powerful (Haslam et al., 1992; Haslam and Turner, 1992, 1995; Parker and Janoff-Bulman, 2013). Doing so may be difficult to do without causing stigma, but at least assessing the impact of these messages is still useful as messages from various countries already blame others residents (Porumbescu et al., 2020). Future research should compare the effects of blame messages against a social identity approach. From a political science perspective (the theory of games), alternative approaches to understanding blame as a functional part to maintaining power may also add to understanding why leaders blame as well (Wagner, 1986). This is to say that we have highlighted one approach to understanding blame, but there are others that might be worth considering.

## Conclusion

It has become clear in the past year that public health officials are fighting two epidemics: an epidemic of COVID-19, and an epidemic of faulty/malice filled information (Islam et al., 2020). This dual burden that COVID-19 represents is likely to continue to affect society for the next decade. At the time of writing, vaccines are being rolled out across the United Kingdom and the world; however, intragroup distrust (as a consequence of being blamed) may impact the roll out. Early surveys suggested ethnic minorities and low income individuals in the United Kingdom will resist getting the vaccine, possibly due to a general distrust of the government (Bell et al., 2020; Dickerson et al., 2021; Razai et al., 2021), which also blamed them (British Broadcasting Corporation [BBC], 2020b). The gap between the rich and the poor is likely to grow as a result of COVID-19 (Adams-Prassl et al., 2020), and ethnoreligious tensions appear to have worsened in some countries (Ide, 2021). Blame in such an environment is especially toxic as it further separates people, when unity is needed against the COVID-19 threat and beyond (Jakovljevic et al., 2020).

Although we have detailed a theoretical account of COVID-19 and blame, precious little literature has attempted to understand how blame works against a theoretical model in COVID-19. This means there are key gaps in our knowledge. Perhaps most notably, there is little experimental evidence that manipulates the conditions of blame in COVID-19, suggesting more research is needed to examine the causality of blame in COVID-19. Understanding how blame functions in COVID-19 is crucial to ensure the recovery from the pandemic occurs evenly, and effectively. Pandemics can result in ethnic tensions when particular groups are blamed, and can even cause further health problems through distrust in systems. As blame can be damaging to a society already ravaged by COVID-19, we must seek to understand blame further through research in this context. In the meantime, avoiding blame as much as possible is critical to ensuring that a post-COVID-19 society is at least as healthy and harmonious as pre-pandemic levels.

## Data Availability Statement

The original contributions presented in the study are included in the article/supplementary material, further inquiries can be directed to the corresponding author/s.

## Author Contributions

AB and VT conceptualized the contribution. AB produced the first draft. VT and CW reviewed the manuscript and provided the critical revision processes. All authors approved the submission of the final version of the manuscript.

## Conflict of Interest

The authors declare that the research was conducted in the absence of any commercial or financial relationships that could be construed as a potential conflict of interest.

## Publisher’s Note

All claims expressed in this article are solely those of the authors and do not necessarily represent those of their affiliated organizations, or those of the publisher, the editors and the reviewers. Any product that may be evaluated in this article, or claim that may be made by its manufacturer, is not guaranteed or endorsed by the publisher.
